# Human Papillomavirus Vaccination Rates in Patients Living With Human Immunodeficiency Virus (HIV)

**DOI:** 10.7759/cureus.57558

**Published:** 2024-04-03

**Authors:** Nicholas Boivin, Samit Desai

**Affiliations:** 1 Infectious Disease, Hackensack Meridian School of Medicine, Nutley, USA; 2 Infectious Disease, Hackensack University Medical Center, Hackensack, USA

**Keywords:** hpv-associated malignancy, hiv disease, cancer in hiv patients, hpv vaccines, hpv vaccination, hpv infection, vaccine, vaccination rate, hpv, hiv

## Abstract

Introduction: Human immunodeficiency virus (HIV) and human papillomavirus (HPV) have a strong association with one another, including the development of HPV-related neoplasms. The Centers for Disease Control and Prevention (CDC) recommends routine HPV vaccination in persons aged 9-26, and consideration can be made to vaccinate up to age 45 based on provider discretion. This study aimed to look at the rate of HPV vaccination in adult HIV-positive patients.

Materials and methods: This was a retrospective study looking at 71 current patients of an HIV clinic at Hackensack University Health. The entire clinic patient list was included. Exclusion criteria were anyone under age 18. Chart review and calls to the patient's pharmacy were done to record the patient's HPV vaccination history. From there, the number of patients eligible to receive the HPV vaccine was calculated based on routine schedule as well as increasing the eligible age up to 44.

Results: Only three patients had a history of receiving the HPV vaccine (4.23%). Using the routine vaccination schedule, there were five patients eligible to receive the HPV vaccine (7%). When using the extended vaccination schedule up to age 44, there were a total of 35 patients eligible to receive the HPV vaccine (49.30%).

Conclusion: There are a substantial number of HIV-positive patients who would benefit from HPV vaccination. This is especially true if the provider chooses to use the extended vaccination schedule. Providers working with HIV-positive patients should probe about vaccination history and intervene as appropriate.

## Introduction

Currently, the Centers for Disease Control and Prevention (CDC) recommends standard human papillomavirus (HPV) vaccination for persons aged 9-26, as well as for persons up to age 45 based on special considerations [1]. Some factors that may warrant extending the vaccination range include if the patient is immunocompromised and the patient's sexual history. Of note, the prevalence of HPV infection is higher in patients who also are infected with the human immunodeficiency virus (HIV) than in HIV-negative persons [2]. Not only is coinfection between these two viruses common, but HPV-related neoplasms have been shown to be present at a higher rate in HIV-positive patients than HIV-negative patients. In fact, a study among North American women suggested an incidence of invasive cervical carcinoma that increases as the patient's CD4 cell count decreases, with an overall incidence of 26 women per 100,000 person-years in the HIV-positive group compared to six women per 100,000 person-years in the HIV-negative group. Another HPV-related cancer, anal squamous cell carcinoma, has been shown to have a prevalence 60-80 times higher in the HIV-positive population than the HIV-negative population [3]. Patients with CD4 count below 200 have also been shown to have an increased rate risk of developing anal cancer [4].

The HPV vaccine has been shown to be safe and effective in numerous studies, allowing it to be a tool to stop the development of cancer especially in susceptible populations such as persons with HIV. Data from the CDC on the Gardasil 9 vaccine has shown an adverse event rate of about 0.026%, and of those, only about 3% are labeled as serious adverse events [5]. There have been a number of studies showing that the HPV vaccine lowers the rate of infection. A recent systematic review has shown the vaccine to be highly effective, primarily when administered to adolescents aged 10-14 [6]. One study showed the prevalence of the four primary oncogenic HPV strains (6, 11, 16, and 18) was reduced by over 80% in women aged 14-24 in the United States from the year 2003 to the year 2015 [7]. Although the vaccination is primarily to prevent primary HPV infection, there is some evidence that it may be beneficial in preventing disease recurrence and/or disease burden in patients already infected [8].

Currently, there are no firm recommendations for the administration of the HPV vaccine in patients over age 26 with HIV. While there have been some questions about the effectiveness of the vaccine in patients with high viral load and/or low CD4 count, due to the high risk of HPV-related neoplasms in HIV-positive patients, it would be reasonable to vaccinate in this population up to age 45 based on the CDC special considerations [9]. There have been few studies looking at the HPV vaccination rate in HIV-positive patients, in particular studies that include patients over age 26. The objective of this study was to evaluate the HPV vaccination rate in HIV-positive patients.

## Materials and methods

Patients

This study was approved by the Hackensack Meridian Health Institutional Review Board (approval number: Pro2023-0130). The requirement for obtaining consent was waived due to the retrospective nature of the study. Hackensack Meridian has a large population of HIV-positive patients who receive their care at the health system's infectious disease clinic. Of these, a subset of patients receive care at the Ryan White clinic which specializes in HIV management. All patients who attend the Ryan White clinic have a diagnosis of HIV confirmed with HIV nucleic acid amplification testing or HIV antibody differentiation immunoassay testing. A universal sampling of the Ryan White clinic patient population was employed, yielding 71 patients who were included in this study.

Of the 71 patients, 18 were female (25.4%) and 53 were male (74.6%). Patients' ages ranged from 21 years to 69 years. The mean age was 42.7 years, and the median age was 43 years. Patients were divided into two age groups: 18-26 years to represent patients eligible for routine HPV vaccination and 18-44 years to represent patients eligible for HPV vaccination using an extended schedule. There were seven patients aged 18-26 years (9.9%), and there were 38 patients aged 18-44 years (52.1%). Baseline characteristics are summarized in Table 1 below.

**Table 1:** Patient baseline characteristics

	Number of patients	Percent of patients (%)
Total sample	71	100
Male patients	53	74.6
Female patients	18	25.4
Patients aged 18-26 years	7	9.9
Patients aged 18-44 years	38	52.1

Analysis

Once the patient list was created, data was gathered using an Excel spreadsheet. Two separate Excel spreadsheets were created in order to help maintain patient anonymity. The first spreadsheet contained the patient's name, medical record number (MRN), and an assigned identification number from 1 to 71 with no repeats. The identification number was assigned alphabetically based on the patient's last name. A sample of what the first spreadsheet appeared as using fictitious patient information is shown below in Table 2.

**Table 2:** Sample of spreadsheet 1 using fictitious patient information MRN: medical record number

Patient name	ID	MRN
Smith, John	55	123456789

The second spreadsheet contained empty columns to fill in with patient information. This included patient identification number, patient gender, patient age, if the patient has a history of prior HPV vaccination, if the patient has a history of HPV infection, if the patient is eligible to receive their vaccine today using routine criteria of the patient being aged 9-26, if the patient is eligible to receive their vaccine today using extended criteria of the patient being aged 9-44, patient's pharmacy name, patient's pharmacy phone number, if the pharmacy was called, and if the pharmacy had recorded a history of patient HPV vaccination not found on chart review. Of note, patient name and MRN were not recorded on this spreadsheet. Patients were deemed eligible to receive the HPV so long as they were in the given age range and had no history of HPV vaccination in the past.

By matching patient identification numbers between the two spreadsheets, information was filled in through chart review. For any patient under age 45, if they had no record of HPV vaccination, their pharmacy was called to verify if they had any information on their HPV vaccination history. Patients who used the clinic pharmacy did not have their pharmacy called as the clinic pharmacy's records would automatically populate during chart review. The patient's primary pharmacy and contact information was discovered through chart review. Pharmacies were called a maximum of three times before being listed as non-responders. A sample of what the second completed spreadsheet looks like using fictitious patient data is included in Table 3 below.

**Table 3:** Sample of spreadsheet 2 using fictitious patient information HPV: human papillomavirus

ID	Gender	Age	Prior HPV vaccination	Prior HPV infection	Eligible for vaccine (9-26 years old)	Eligible for vaccine (9-44 years old)	Pharmacy name	Pharmacy phone number	Pharmacy called	New HPV history from the pharmacy
17	M	27	No	No	No	Yes	XXX	222-222-2222	Yes	No

Once all the spreadsheet cells were filled in, a manual count was done on how many patients had received their HPV vaccination, how many pharmacies were called, how many patients had HPV vaccination records from their pharmacy not found on the chart review, how many patients were eligible for vaccination in the 18-26-year-old group, how many patients were eligible for vaccination in the 18-44-year-old group, and how many patients had a history of HPV infection. In addition, out of the patients in the 18-44-year-old group, the number of patients who met the criteria for a pharmacy call but were using the clinic's on-site pharmacy was recorded.

From there, basic math was performed to find the percentage of patients in the total sample population (as well as within the two age groups) who were eligible to receive their HPV vaccination. Basic math was also done to find the percentage of patients with a history of HPV infection in the total sample, and then this was divided by gender.

## Results

Of the 71 total patients, only three had a history of receiving the HPV vaccine (4.23%). There were a total of 44 patients who met the initial criteria to have their pharmacy called based on vaccination history and age; however, 11 out of the 44 patients used the clinic's on-site pharmacy (25%). Pharmacies were contacted about HPV vaccination records for the remaining 33 patients; of these, four pharmacies were non-responders.

No pharmacy had any additional records for any patient. There were three patients who did not have a pharmacy listed on the chart. Of these, one patient was eligible to receive the vaccine using the routine criteria, and all three were eligible to receive the vaccine using the extended criteria.

Out of the seven patients aged 18-26 years, two of them had a history of receiving the HPV vaccine, leaving five patients within the age group eligible for HPV vaccination based on routine schedule (71.4%). Thus, out of the total sample of 71 patients, there were five patients eligible for HPV vaccination using the routine schedule (7%).

Out of the 38 patients aged 18-44 years, three of them had a history of receiving the HPV vaccine, leaving 35 patients within the age group eligible for HPV vaccination based on extended scheduling (92.1%). Therefore, out of the total sample of 71 patients, there were 35 eligible for HPV vaccination using an extended scheduling up to age 44 years (49.3%).

There were 14 patients who had an indication of previous HPV infection (19.72%). Of these 14 patients, six were female (42.9%), and eight were male (57.1%). Findings are summarized below in Table 4.

**Table 4:** Vaccination rate, vaccination eligibility, and past HPV infection rate in the patient sample HPV: human papillomavirus

	Number of patients	Percent of patients (%)
Total sample	71	100
Record of previous HPV vaccination	3	4.23
Eligible to receive HPV vaccination using routine schedule	5	7
Eligible to receive HPV vaccination going up to age 44	35	49.3
History of previous HPV infection	14	19.72

## Discussion

This study showed an estimated prevalence of 19.72% in an HIV-positive adult population. It is estimated that the prevalence of oral HPV in the general adult population in the United States is approximately 7.3% and the prevalence of genital HPV in the US adult population is 40-45% [10]. Previous studies have suggested that HIV-positive patients have a higher prevalence of HPV infection than HIV-negative patients [2, 11]. It is likely that this study did not show that connection due to the small sample size and a lack of routine testing in the selected patient group.

Although the number of patients eligible for the HPV vaccine using a routine vaccination schedule was modest, almost half of the total sample of patients were eligible for the vaccine based on the extended schedule of vaccinating up to age 44. In addition, when only considering patients within their respective age groups (18-26-year group and 18-44-year group), the percentage of patients eligible to receive their HPV vaccination was substantial.

The expansion of the HPV vaccination schedule to allow administration up to age 45 is a recent one, occurring in 2019 [12]. The recency of this change, combined with questions of efficacy in an older patient population, is likely why there is no clear consensus on whether HIV-positive patients should receive the HPV vaccine over age 26 [13,14]. Because the vaccine is so safe, even without a strong consensus, it is not unreasonable for a provider to recommend the HPV vaccine to an HIV-positive patient who is aged 27-45. One of the main deterrents to vaccinating in this older age group to consider is the cost. Some insurance companies may not cover administration, and with a list price of close to $300 for Gardasil 9, the cost-benefit may not be appropriate for some patients [15].

Regardless of whether the provider chooses to follow the routine vaccination schedule or the extended schedule, this study shows that within the given age parameters, there is a high percentage of HIV-positive patients who would be able to receive their HPV vaccination. Besides asking about vaccination history during office visits, pharmacies can also evaluate vaccination history and intervene as appropriate. Pharmacists have noted multiple barriers to administering the HPV vaccine including getting insurance reimbursement, difficulty completing the vaccination series, difficulty keeping track of all patients, and more [16]. Providers can alleviate some of this burden by writing prescriptions for the HPV vaccination and helping to make sure patients are completing their vaccination series by making notes in the patient chart of when the next dose in the series should be given.

This study had several limitations. The first limitation is the small sample size limiting generalizability and introducing potential sampling error. The patient population being taken from only one clinic also limits the generalizability of the study. Another limitation is that all data was garnered from chart review. Face-to-face discussions with patients may have revealed differing information. This may mean that the results of the study may not be fully accurate. The main strengths of this study are that two sources of vaccination history were investigated to acquire data and this study is one of the first to evaluate HPV vaccination rates in HIV-positive patients over the age of 26.

## Conclusions

This study shows many patients who are HIV-positive have a low rate of HPV vaccination. There are likely many HIV-positive patients who are eligible to receive their HPV vaccination currently attending clinics, presenting an opportunity for provider intervention. This is especially true if the provider chooses to follow the extended vaccination schedule up to age 45. The lack of HPV vaccination history in HIV-positive patients demonstrated in this study suggests that providers should inquire about vaccination history in all HIV-positive patients and intervene as necessary.
